# Molecular Detection of Filarial and Avian Haemosporidian Parasites in *Culicoides* Biting Midges (Diptera: Ceratopogonidae) from Laos

**DOI:** 10.3390/insects17090963

**Published:** 2026-09-18

**Authors:** Ronnalit Mintara, Wannachai Wannasingha, Chavanut Jaroenchaiwattanachote, San Namtaku, Khamla Inkhavilay, Isara Thanee, Pairot Pramual

**Affiliations:** 1 Department of Biology, Faculty of Science, Mahasarakham University, Mahasarakham 44150, Thailand; ronnalitmintara@gmail.com (R.M.); isara.th@msu.ac.th (I.T.); 2 Center of Excellence in Biodiversity Research, Mahasarakham University, Mahasarakham 44150, Thailand; wannachai.w@msu.ac.th; 3 Biomedical Science Research Unit, Mahasarakham University, Mueang Mahasarakham 44000, Thailand; chavanut.j@msu.ac.th; 4 Department of Science and Mathematics, Faculty of Science and Health Technology, Kalasin University, Kalasin 46230, Thailand; 5 Center of Excellence in Biodiversity, National University of Laos, Vientiane 7322, Laos; khamla.inkhavilay@nuol.edu.la

**Keywords:** biting midges, insect vector, vector-parasite association

## Abstract

Species of *Culicoides* Latreille (Diptera: Ceratopogonidae) are important biting pests and vectors of pathogens affecting humans and animals. However, information on *Culicoides*–parasite associations in Laos is lacking. We used molecular methods to screen 237 specimens representing 27 *Culicoides* species from Laos for filarial nematodes, avian haemosporidians, *Trypanosoma* spp., and *Leishmania* spp. Overall, 59 specimens representing 19 *Culicoides* species were positive for at least one parasite. Only filarial nematodes and avian haemosporidians were detected. Filarial parasites were found in four *Culicoides* species (*C. actoni*, *C*. *guttifer*, *C. huffi*, and *C. mahasarakhamense*). Analysis of 12S rRNA sequences revealed at least two filarial taxa, one closely related to *Onchocerca lienalis* and the other to an unidentified onchocercid previously detected in *Culicoides*. Avian haemosporidians were more prevalent. *Leucocytozoon* spp. were detected in 40 specimens from 18 *Culicoides* species, with *cyt b* sequences identifying *Leucocytozoon caulleryi* and an unidentified *Leucocytozoon* sp., the latter accounting for 39 of the 40 positive specimens. *Plasmodium juxtanucleare* was detected in 27 specimens (11.4%), whereas *Haemoproteus* spp. were found in only two specimens (0.8%), which represented two distinct lineages previously reported from wild birds. No *Trypanosoma* or *Leishmania* DNA was detected. *Culicoides mahasarakhamense* and *C. huffi* had the highest infection frequencies. Their high abundance and documented feeding on livestock, particularly chickens and cattle, suggest that they may play important roles in pathogen transmission and should be prioritized for future vector surveillance in Laos.

## Simple Summary

Insect-borne diseases are major threats to human and animal health. Understanding which blood-feeding insects carry parasites is essential for assessing disease risk and developing effective control strategies. In this study, we screened biting midges of the genus *Culicoides* from Laos for parasites using molecular methods. Overall, 59 of 237 female specimens (25%) were infected with at least one parasite. The detected parasites belonged to the groups Onchocercidae (filarial nematodes), *Leucocytozoon* spp., *Plasmodium* sp., and *Haemoproteus* spp. Several abundant *Culicoides* species (*C. actoni*, *C. mahasarakhamense*, *C. huffi*, and *C. guttifer*) are known to feed on livestock, including chickens, cattle, and water buffalo, which are potential hosts of these parasites. Our findings provide the first molecular evidence of parasite infections in *Culicoides* spp. from Laos and identify several species that may contribute to parasite transmission in livestock. These results provide a foundation for future studies on the epidemiology and control of insect-borne diseases in the country.

## 1. Introduction

Biting midges of the genus *Culicoides* Latreille (Diptera: Ceratopogonidae) comprise approximately 1360 described species worldwide [1,2]. Many species are important pests and biological vectors that transmit a wide range of pathogens, including viruses, bacteria, protozoa, and filarial nematodes, to humans and other vertebrates [3]. Major diseases transmitted by *Culicoides* spp. include Oropouche fever and mansonellosis in humans, bluetongue disease in ruminants, African horse sickness in equids, and leucocytozoonosis in domestic poultry and wild birds [3]. Outbreaks of these diseases can result in substantial economic losses to the livestock industry. For example, bluetongue disease is estimated to cause annual global economic losses of approximately USD 3 billion [4]. Likewise, the 2020 outbreak of African horse sickness in Thailand affected more than 2700 horses and resulted in the deaths of 565 animals [5,6]. These examples highlight the substantial medical, veterinary, and economic importance of *Culicoides* spp. worldwide.

Birds are also affected by several groups of vector-borne blood parasites, particularly avian haemosporidians of the genera *Plasmodium*, *Haemoproteus*, and *Leucocytozoon*, which are globally distributed and infect a wide diversity of wild and domestic birds. Their prevalence and diversity can vary considerably among host species and geographic regions and can be influenced by host age, environmental conditions, and habitat characteristics [7,8,9,10,11]. Although many infections are subclinical, avian haemosporidians can negatively affect host health and, in some circumstances, cause severe or fatal disease, particularly in susceptible hosts [12] (Yoshimoto et al., 2021). *Culicoides* spp. are particularly important vectors of *Haemoproteus* spp. of the subgenus *Parahaemoproteus* and have also been implicated in the transmission of *Leucocytozoon* spp. in birds. Consequently, understanding associations between biting midges and avian blood parasites is important for assessing their potential effects on poultry production, captive birds, and wild bird populations.

To date, 71 *Culicoides* species have been recorded from Laos [13,14]. Among these, adult females of at least four species (*C. fulvus* Sen and Das Gupta, *C. guttifer* (Meijere), *C. peregrinus* Kieffer, and *C. oxystoma* Kieffer) are recognized vectors of medically and veterinary-important pathogens [3]. Several additional species reported from Laos are considered potential vectors because they have been found carrying pathogenic organisms. In neighboring Thailand, for example, avian haemosporidians (*Leucocytozoon* spp. and *Plasmodium* spp.) and *Trypanosoma* spp. have been detected in *C. huffi* Causey and *C. mahasarakhamense* [15,16,17,18]. Bluetongue virus has also been detected in *C. fulvus*, *C. orientalis* Macfie, and *C. oxystoma* [19]. Furthermore, the human pathogenic protozoa *Leishmania martiniquensis* Desbois, Pratlong, and Dedet and *L. orientalis* Bates and Jariyapan, which cause leishmaniasis, have been detected in several *Culicoides* species, including *C. actoni* Smith, *C. arakawae* (Arakawa), *C. fulvus*, *C. guttifer*, *C. innoxius* Sen and Das Gupta, *C. jacobsoni* Macfie, *C. mahasarakhamense*, *C. orientalis*, *C. oxystoma*, *C. peregrinus* Kieffer, *C. shortti* Smith and Swaminath, and *C. sumatrae* Macfie [20,21,22]. Several of these potential vector species, particularly *C. peregrinus*, *C. mahasarakhamense*, and *C. oxystoma*, are abundant in Laos. Blood-meal analyses have shown that these species commonly feed on domestic animals (including chickens), water buffaloes, and cattle, respectively [14], suggesting that their high abundance and feeding preferences may facilitate pathogen transmission to economically important livestock. Despite this potential risk, there is a lack of information on pathogen infections in *Culicoides* populations from Laos, limiting our understanding of disease ecology and hindering the development of effective surveillance and control strategies.

In the present study, we employed PCR-based molecular screening to detect haemosporidians, *Trypanosoma* spp., *Leishmania* spp., and filarial nematodes in *Culicoides* spp. collected from Laos. Although molecular detection of pathogen DNA in hematophagous insects does not, by itself, demonstrate vector competence [23,24], it provides valuable baseline information for identifying candidate vector species and prioritizing them for subsequent investigations of vector competence and pathogen transmission [25]. Because some closely related *Culicoides* species are morphologically similar and difficult to distinguish based solely on morphological characteristics [16], we used an integrative approach combining morphological and molecular methods for species identification.

## 2. Materials and Methods

### 2.1. Culicoides Specimens and Species Identification

**Figure 1:**
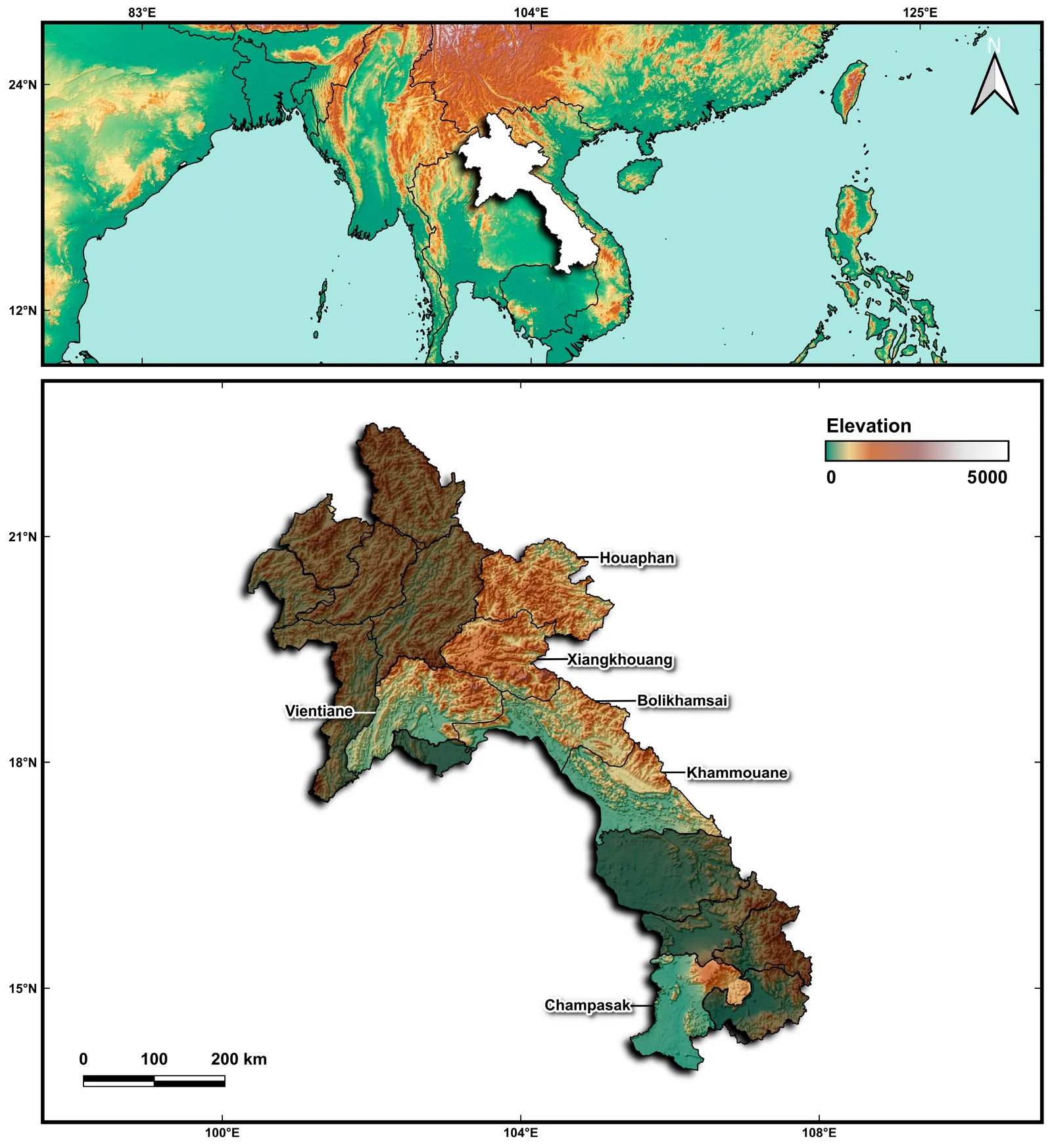
Map showing the geographical location of Laos (white area in the top panel) and the *Culicoides* sampling areas in six provinces of Laos (bottom panel). The map of Laos was retrieved from the Humanitarian Data Exchange (HDX) (https://data.humdata.org/dataset/cod-ab-lao) (accessed on 18 March 2026), and topographic data were obtained from the EarthEnv project (https://www.earthenv.org/topography) (accessed on 18 March 2026).

Female *Culicoides* specimens collected during our previous survey of biting midges in Laos [14] were used for molecular screening of parasites. The specimens were collected between August 2024 and October 2025 from six provinces of Laos: Vientiane, Borikhamxai, Houaphan, Khammouan, Xaisomboun, and Xiangkhoang (Figure 1). Sampling was conducted during this period, which encompassed the rainy seasons, when *Culicoides* populations are generally more abundant, thereby increasing the likelihood of obtaining sufficient specimens for parasite screening. Detailed information on sampling methods and collection sites has been reported previously [14]. We included all *Culicoides* species for which female specimens were available from our collections. For species collected at more than one sampling location, specimens from each available location were selected to provide broader geographic representation. Specimens were initially identified morphologically using the taxonomic keys and species descriptions of *Culicoides* spp. from Laos [13], Southeast Asia [26], and the *Culicoides* spp. Wing Atlas [27]. The morphological parts of the specimens (heads, wings, and legs) retained for species identification were preserved in 80% ethanol and stored at −20 °C in the Insect Biodiversity Laboratory, Department of Biology, Faculty of Science, Mahasarakham University, Thailand, for future reference and study. For specimens that tested positive for parasites, species identifications were further confirmed by DNA barcoding of the mitochondrial cytochrome c oxidase subunit I (COI) gene.

### 2.2. DNA Extraction, Parasite Detection, and Sequencing

DNA was extracted from the whole individual adult female specimens using the GF-1 Nucleic Acid Extraction Kit (Vivantis Technologies Sdn. Bhd., Subang Jaya, Malaysia) according to the manufacturer’s instructions. Extracted DNA was stored at −20 °C until further analysis. For molecular confirmation of *Culicoides* species, a fragment of the mitochondrial COI gene was amplified using the universal primers LCO1490 and HCO2198 [28] following the PCR protocol described by Tangkawanit et al. [29].

Filarial parasites were detected by amplifying an approximately 550 bp fragment of the 12S rRNA gene using the primers 12SOvC and 12SOvB with PCR conditions described by Morales-Hojas et al. [30]. DNA extracted from a filarial parasite previously detected in *Culicoides* sp. from Thailand [17] was used as a positive control. Haemosporidian parasites were screened using the nested PCR protocol of Hellgren et al. [31]. The first-round PCR employed the primers HaemNFI and HaemNR3 to amplify a fragment of the mitochondrial cytochrome *b* (*cyt b*) gene. The second-round PCR used the primers HaemFL and HaemR2L for the specific detection of *Leucocytozoon* spp., whereas the primers HaemF and HaemR2 [32] were used to detect *Plasmodium* and *Haemoproteus*. PCR conditions followed Jumpato et al. [33]. DNA extracted from haemosporidian parasites previously detected in black flies (*Simulium* spp.) from Thailand [27] was used as positive controls. Detection of *Leishmania* was performed by amplifying an approximately 379 bp fragment of the internal transcribed spacer 1 (ITS1) region of the ribosomal RNA gene using the primers LeF and LeR [28] under the PCR conditions described by Spanakos et al. [34]. *Trypanosoma* was detected using the primers S-762 and S-763 [35], which amplify an approximately 2000 bp fragment of the small subunit ribosomal RNA (SSU rRNA) gene. PCR amplification followed the protocol described by Thaijarern et al. [36]. DNA extracted from *Trypanosoma* sp. previously detected in *Simulium* spp. from Thailand [36] was used as positive controls. For all PCR reactions, a negative control containing sterile distilled water instead of template DNA was included to monitor for potential contamination.

PCR products were visualized by electrophoresis on 1% agarose gels stained with Novel Juice (GeneDirex, Taoyuan, Taiwan, China). Successfully amplified products were purified using the GF-1 AmbiClean (Gel & PCR) Kit (Vivantis Technologies Sdn. Bhd., Subang Jaya, Malaysia) and sequenced bidirectionally at ATCG Company Limited (Thailand Science Park, Pathum Thani Province, Thailand) using the same primers employed for PCR amplification.

### 2.3. Data Analysis

Sequence chromatograms (Supplementary Data S1) were inspected manually and edited using the Edit/View Sequence function in MEGA version X [37]. A total of 24 COI sequences of *Culicoides* spp. were generated in this study (GenBank accession nos. PZ798657-PZ798680). In addition, six 12S rRNA sequences of filarial parasites (GenBank accession nos. PZ815588-PZ815593) and 69 *cyt b* sequences of haemosporidian parasites (GenBank accession nos. PZ809999-PZ810067) were obtained. No *Leishmania* or *Trypanosoma* DNA was detected in any of the *Culicoides* specimens examined.

Species identification of *Culicoides* spp. based on COI barcodes was performed using the Identification Engine of the Barcode of Life Data System (BOLD database (https://boldsystems.org/), employing the Animal Species-Level Library (Public + Private) and the Rapid Species Search option [38]. Parasite sequences were identified by comparison with sequences available in GenBank using the Basic Local Alignment Search Tool (BLAST) (https://blast.ncbi.nlm.nih.gov/Blast.cgi) (accessed on 3 March 2026). Phylogenetic analyses were performed using both Neighbor-Joining (NJ) and Maximum Likelihood (ML) methods implemented in MEGA version X [37] to determine the genetic relationships of parasite sequences detected in *Culicoides* spp. from Laos with reference sequences retrieved from GenBank based on BLAST searches.

For filarial parasites, a total of 33 sequences, including the six sequences generated in this study, were included in the phylogenetic analyses. Haemosporidian parasites were analyzed separately for *Leucocytozoon* spp. and *Haemoproteus* spp. The analyses included 40 and 2 sequences generated in this study, together with 40 and 30 reference sequences retrieved from GenBank for *Leucocytozoon* spp. and *Haemoproteus* spp., respectively. Phylogenetic analyses were not performed for *Plasmodium* because all detected in *Culicoides* spp. from Laos were *P. juxtanucleare*.

Neighbor-Joining trees were constructed using the Kimura two-parameter (K2P) model, and node support was assessed with 1000 bootstrap replicates. Maximum Likelihood analyses were performed using the General Time Reversible model with a proportion of invariant sites and gamma-distributed rate variation among sites (GTR + I + G). Branch support was estimated using the ultrafast bootstrap method with 1000 replicates.

## 3. Results

### 3.1. DNA Barcoding of Culicoides Species Positive for Parasites

A total of 237 adult female specimens representing 27 *Culicoides* species were included in this study (Table 1). In total, 59 *Culicoides* specimens tested positive for at least one parasite (Table 1). COI sequences of 25 specimens of *Culicoides* from Laos were reported previously [8], whereas 24 additional sequences (GenBank accession nos. PZ798657-PZ798680) were generated in the present study. Identification using the Barcode of Life Data System (BOLD) showed that 21 of the 24 newly generated sequences were congruent with morphological identification and included *C. huffi* (*n* = 11), *C. mahasarakhamense* (*n* = 9), and *C. peregrinus* (*n* = 1). Three specimens showed discrepancies between morphological and molecular identifications. Two specimens morphologically identified as *C. mahasarakhamense* were assigned by DNA barcoding to *C. arakawae* and *C. thurmanae*. Likewise, one specimen morphologically identified as *C. huffi* was identified as *C. similis* based on its COI sequence.

**Figure 2:**
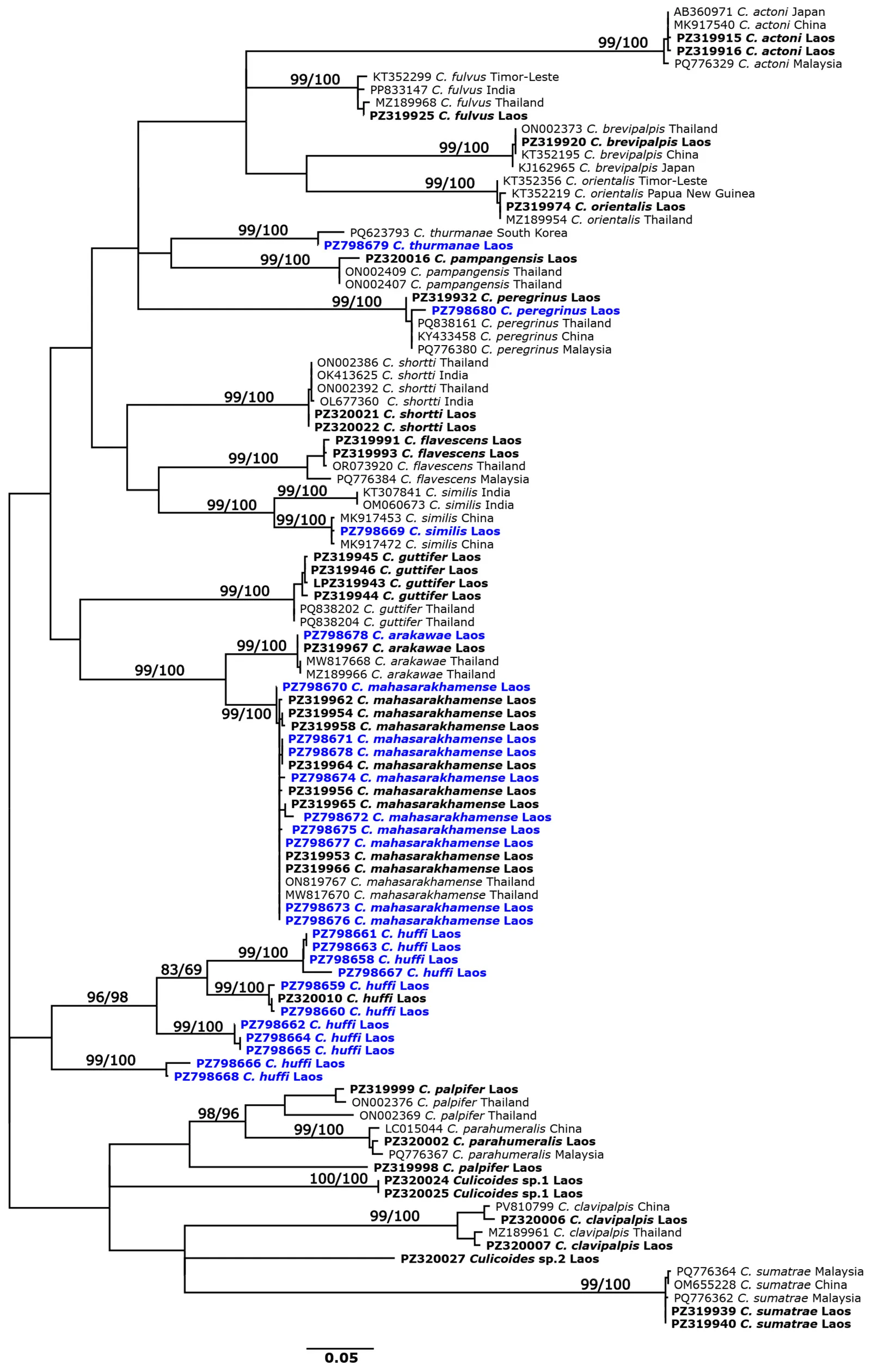
Maximum likelihood tree based on 102 mitochondrial cytochrome oxidase I gene sequences, including 59 from Laos (24 generated in this study (blue)), that were infected with parasites (bold), representing 21 *Culicoides* species. For each species, the country of origin and NCBI GenBank accession number are provided. Bootstrap values for ML and NJ analyses are shown above or near the branches.

Phylogenetic analyses based on NJ and ML methods resolved similar tree topologies; therefore, only the latter is presented (Figure 2). All species are monophyletic with strong bootstrap support (>99%) except for *C. palpifer*, as one specimen separated from the main clade.

### 3.2. Filarial Parasites in Culicoides spp. from Laos

Filarial parasites were detected in six specimens representing four *Culicoides* species: *C. actoni* (*n* = 1), *C. guttifer* (*n* = 2), *C. huffi* (*n* = 2), and *C. mahasarakhamense* (*n* = 1) (Table 2). Comparison of the 12S rRNA sequences with those available in GenBank revealed that five sequences (one from *C. mahasarakhamense* and two each from *C. guttifer* and *C. huffi*) shared >99% sequence identity with an unidentified Onchocercidae previously reported from *C. mahasarakhamense* in northeastern Thailand [11]. The remaining sequence, detected in *C. actoni*, was most closely related (98% sequence identity) to *Onchocerca lienalis* (GenBank accession no. AY462925) from cattle in the UK (Table 2).

**Figure 3:**
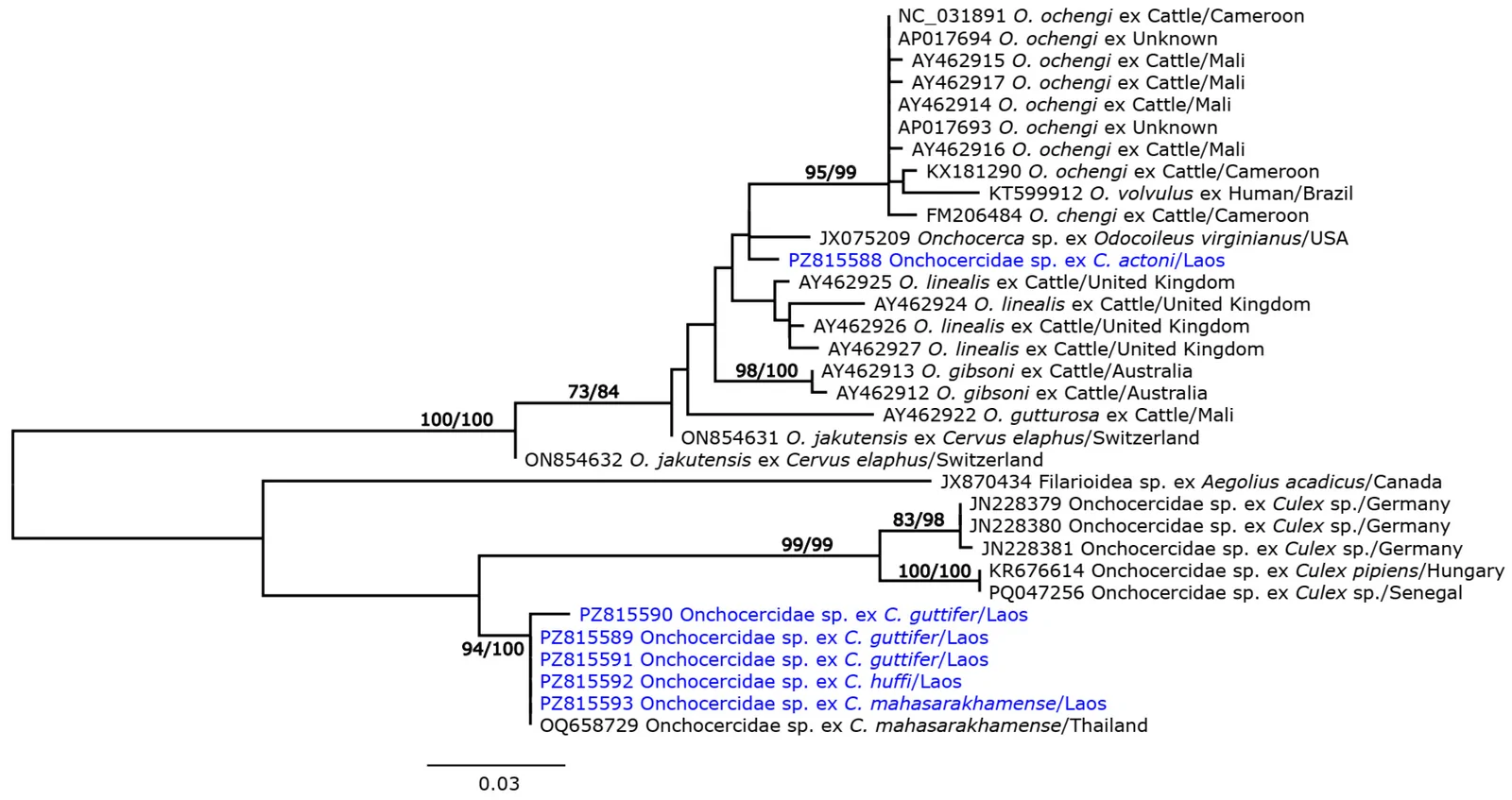
Maximum likelihood tree based on the mitochondrial 12S rRNA gene sequences of six filarial parasites detected in *Culicoides* species in Laos (blue) and closely related sequences retrieved from GenBank. Bootstrap values (1000 replicates) for ML and NJ are above the branches. Names of vertebrate hosts or invertebrate vectors and country of origin (if available in GenBank) are listed following the accession number.

Neighbor-Joining (NJ) and Maximum Likelihood (ML) analyses produced similar phylogenetic topologies; therefore, only the ML tree is presented (Figure 3). The phylogeny resolved two major clades. Clade I comprised *Onchocerca* species, including *O. lienalis*, *O. gutturosa*, *O. fasciata*, *O. jakutensis*, *O. gibsoni*, *O. ochengi*, *O. volvulus*, *O. lupi*, *O. flexuosa*, and *Onchocerca* sp. The filarial parasite detected in *C. actoni* was placed within this clade and clustered with *O. lienalis* from several geographic regions. Clade II consisted of unidentified Onchocercidae detected in five specimens from Laos (two each from *C. guttifer* and *C. huffi* and one from *C. mahasarakhamense*), which formed a strongly supported cluster (98% bootstrap support) with unidentified Onchocercidae previously detected in *C. mahasarakhamense* from Thailand. Additional members of this clade included unidentified Onchocercidae reported from *Culex* spp. in Germany and Senegal, *Culex pipiens* in Hungary, and *Filarioidea* sp. from *Aegolius acadicus* in Canada.

### 3.3. Haemosporidian Parasites in Culicoides spp. from Laos

Haemosporidian parasites were detected in 55 specimens representing 21 *Culicoides* spp. The most frequently detected parasites were *Leucocytozoon* spp. (40 of 55 positive specimens), followed by *Plasmodium* spp. (27 of 55), whereas *Haemoproteus* spp. were detected in only two specimens. Mixed infections were detected in 14 specimens, including 13 coinfections with *Leucocytozoon* spp. and *Plasmodium* spp. and one coinfection with *Leucocytozoon* sp. and *Haemoproteus* sp.

#### 3.3.1. *Leucocytozoon* spp.

**Figure 4:**
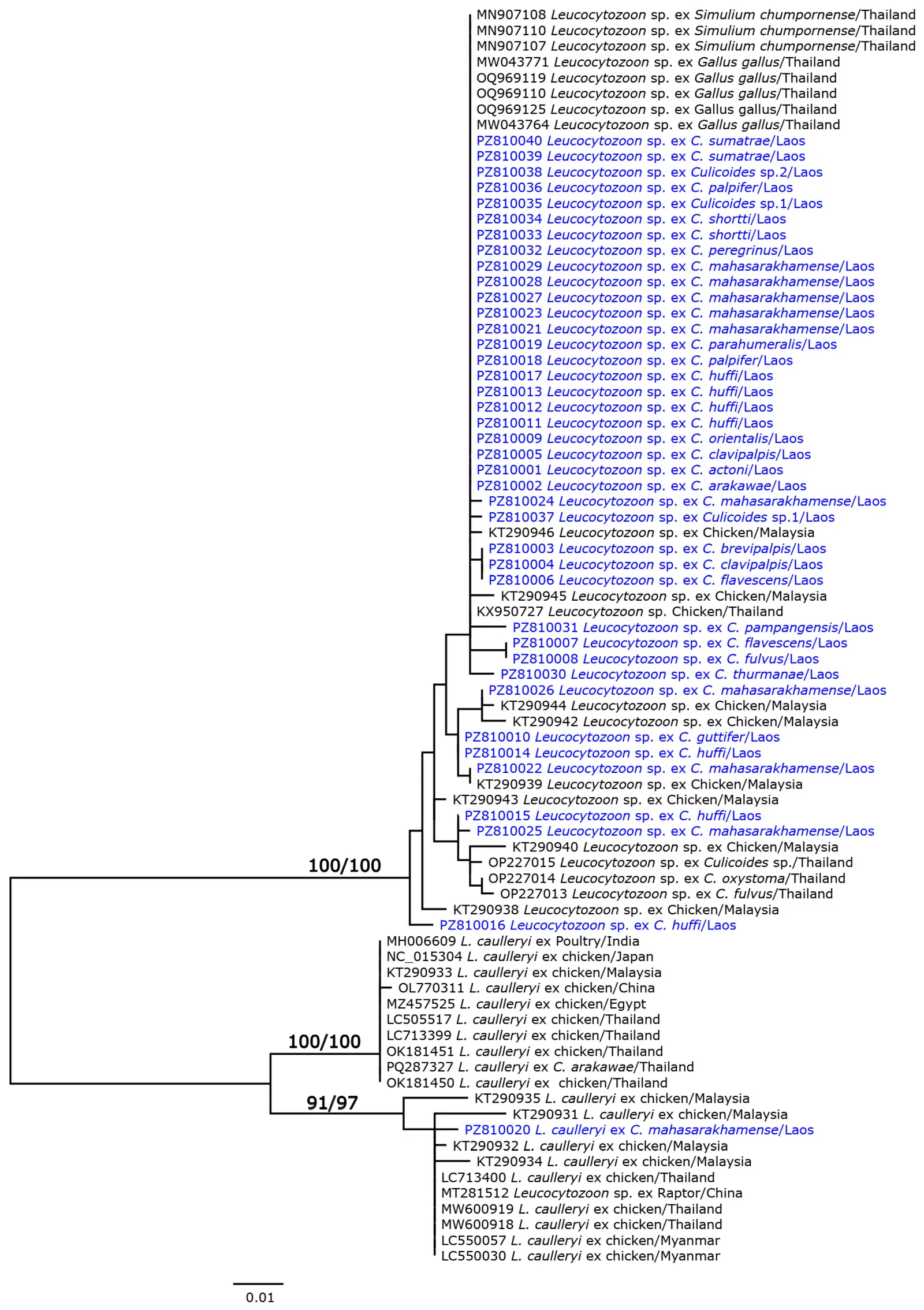
Maximum likelihood tree based on the mitochondrial cytochrome *b* gene sequences of 40 *Leucocytozoon* specimens detected in *Culicoides* species in Laos (blue) and closely related sequences retrieved from GenBank. Bootstrap values (1000 replicates) for ML and NJ are above the branches. Names of vertebrate hosts or invertebrate vectors and country of origin (if available in GenBank) are listed following the accession number.

*Leucocytozoon* spp. were the most frequently detected parasite, occurring in 40 specimens representing 19 *Culicoides* species (Table 1 and Table 3). *Culicoides mahasarakhamense* had the highest number of positive specimens (*n* = 10), followed by *C. huffi* (*n* = 7). The remaining species each contained one or two infected individuals. Sequence comparisons of the cyt *b* gene showed that 39 of the 40 sequences shared >98% identity with unidentified *Leucocytozoon* sp. previously detected in domestic chickens (*Gallus gallus*) from Malaysia and *Culicoides* sp. from Thailand (Table 3). The remaining sequence, detected in *C. mahasarakhamense*, shared >99% identity with *Leucocytozoon caulleryi* previously reported from chickens in Thailand. NJ and ML analyses produced similar phylogenetic trees; therefore, only the ML tree is shown (Figure 4). Two major clades were recovered. Most *Leucocytozoon* sequences from Laos clustered with unidentified *Leucocytozoon* sp. previously reported from chickens and hematophagous insects, including black flies (*Simulium*) and biting midges, from neighboring countries. The second clade consisted of *L. caulleryi* detected in *C. mahasarakhamense* together with reference sequences from chickens in Southeast Asia.

#### 3.3.2. *Plasmodium*

*Plasmodium* was detected in 27 specimens representing six *Culicoides* species (Table 4). The highest numbers of positive specimens were found in *C. mahasarakhamense* (*n* = 11) and *C. huffi* (*n* = 9), followed by *C. guttifer* (*n* = 3), *C. arakawae* (*n* = 2), *C. clavipalpis* (*n* = 1), and *C. peregrinus* (*n* = 1). All cyt *b* sequences were identified as *Plasmodium juxtanucleare*, sharing >99% sequence identity with isolates previously reported from domestic chickens (Table 4).

#### 3.3.3. *Haemoproteus* spp.

**Figure 5:**
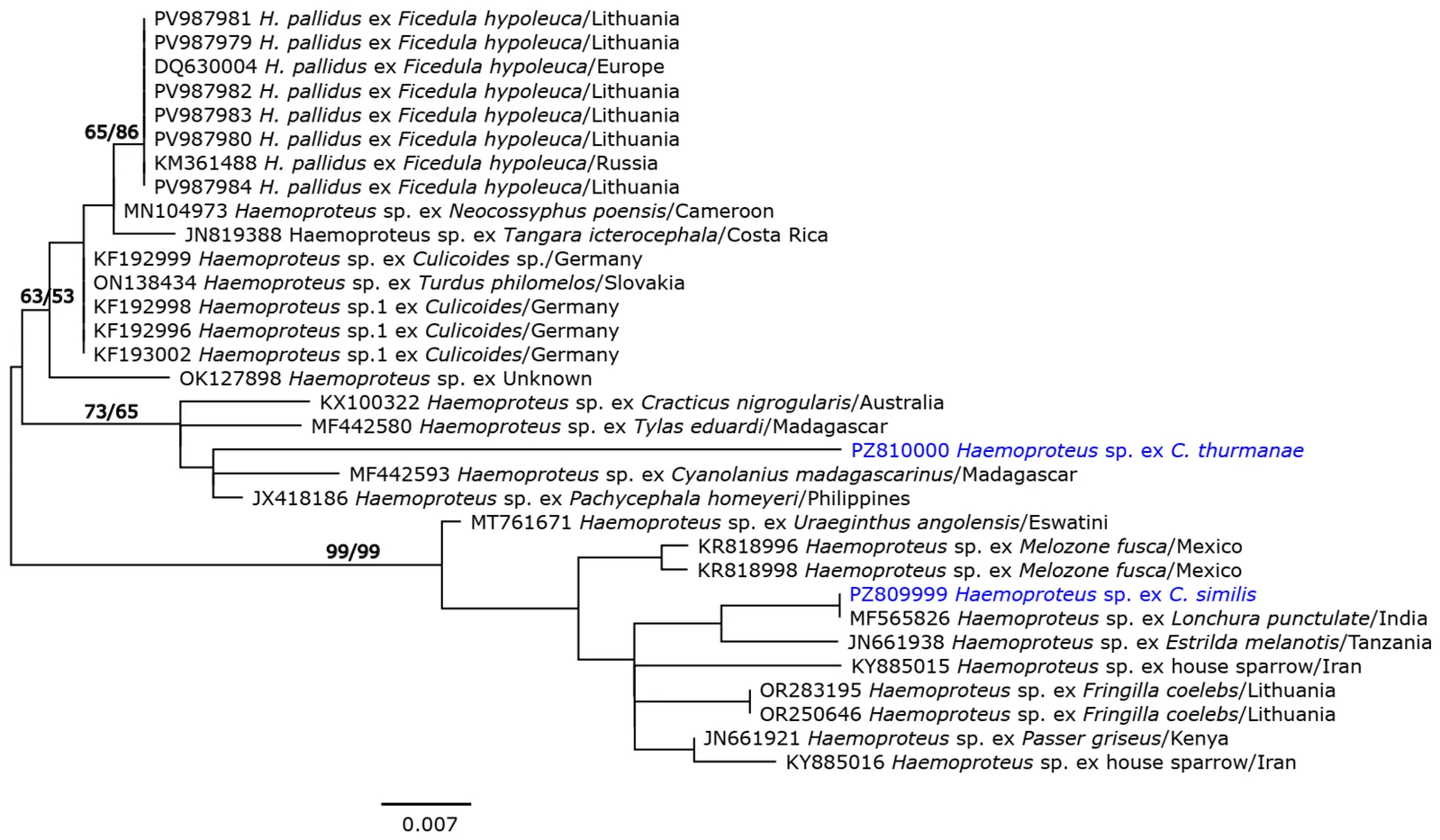
Maximum likelihood tree based on the mitochondrial cytochrome *b* gene sequences of two *Haemoproteus* spp. specimens detected in *Culicoides* species in Laos (blue) and closely related sequences retrieved from GenBank. Bootstrap values (1000 replicates) for ML and NJ are above the branches. Names of vertebrate hosts or invertebrate vectors and country of origin (if available in GenBank) are listed following the accession number.

*Haemoproteus* spp. were detected in two specimens, one each from *C. similis* and *C. thurmanae*. The sequence obtained from *C. similis* was identical to a *Haemoproteus* lineage previously reported from the spotted munia (*Lonchura punctulata*) in India [39]. The sequence from *C. thurmanae* showed 97% similarity to an unidentified *Haemoproteus* species detected in *Culicoides* sp. from Germany [40] (Table 5). NJ and ML analyses produced similar phylogenetic relationships; therefore, only the ML tree is presented (Figure 5). The two *Haemoproteus* spp. sequences from Laos were placed in separate clades, each clustering with lineages previously detected in a variety of avian hosts.

Coinfections involving filarial and haemosporidian parasites were detected in two specimens. One specimen of *C. guttifer* was simultaneously infected with an unidentified Onchocercidae and *Plasmodium juxtanucleare*. One specimen of *C. mahasarakhamense* harbored an unidentified Onchocercidae together with *Leucocytozoon* sp. and *P. juxtanucleare*. Coinfection with *Leucocytozoon* and *Plasmodium* was the most common pattern, occurring in 13 of the 55 haemosporidian-positive specimens, whereas only one specimen exhibited coinfection with *Leucocytozoon* and *Haemoproteus*.

## 4. Discussion

Accurate species identification is fundamental for downstream biological studies, particularly those involving disease epidemiology and insect vectors [41,42,43]. In this study, we demonstrate that an integrative taxonomic approach combining morphological identification with DNA barcoding is essential for accurately assessing the biodiversity of *Culicoides* biting midges. Three specimens showed discrepancies between morphological and molecular identification. One specimen identified morphologically as *C. huffi* was assigned to *C. similis* based on COI sequences, whereas the other two specimens identified as *C. mahasarakhamense* were molecularly identified as *C. arakawae* and *C. thurmanae*. Such discrepancies may result from insufficient genetic variation in the molecular marker or errors in morphological identification [44,45]. Numerous studies have demonstrated that COI sequences provide reliable species discrimination in *Culicoides* [16,46,47,48,49]. Therefore, limited resolution of the COI marker is unlikely to explain these discrepancies. Instead, morphological misidentification is the most plausible explanation. Adult *Culicoides* spp. are extremely small (1–3 mm) and are easily damaged during collection, particularly the wings, which provide many of the diagnostic characters used for species identification. The wing patterns of *C. huffi* closely resemble those of *C. similis*. Similarly, wing patterns of *C. mahasarakhamense* are very similar to *C. arakawae* and *C. thurmanae*. Therefore, increasing the likelihood of misidentification. Consequently, *C. similis* and *C. thurmanae* are reported here for the first time from Laos. Following the checklist of Howarth [13], which documented 66 *Culicoides* species in Laos, and our previous study that added five new country records, the discovery of these two additional species increases the known *Culicoides* fauna of Laos to 73 species.

Biting midges of the genus *Culicoides* are recognized vectors of a wide range of pathogens, including viruses, protozoa, and filarial nematodes [3]. Our findings indicate that several *Culicoides* species in Laos are candidate vector species of both filarial nematodes and haemosporidian parasites. Filarial nematodes were detected in four species (*C. actoni*, *C. mahasarakhamense*, *C. huffi*, and *C. guttifer*). Based on *cyt b* sequences, these parasites were separated into two major groups. The parasite detected in *C. actoni* shared more than 98% sequence similarity with several members of the genus *Onchocerca*. Phylogenetic analysis further placed this parasite within the *O. lienalis* clade. Additional studies are required to determine its species identity, although it is likely to represent a bovine *Onchocerca* species circulating among cattle. *Onchocerca lienalis* is transmitted by black flies, with *Simulium jenningsi* and *S. ornatum* recognized as its principal vectors [50]. The detection of a bovine *Onchocerca* species in *C. actoni* is consistent with the known feeding preference of this biting midge for water buffalo [14].

The second filarial lineage detected in Laos was genetically most similar to an unidentified onchocercid previously reported from *C. mahasarakhamense* in Thailand [17]. Phylogenetic analyses showed that parasites detected in *C. huffi*, *C. guttifer*, and *C. mahasarakhamense* from Laos formed a clade together with those unidentified onchocercid parasites previously reported from mosquitoes and *C. mahasarakhamense* in Thailand. Although the vertebrate hosts remain unknown, the predominantly avian feeding habits of these *Culicoides* species [14,51] indicate that these parasites may infect birds. More than 160 filarioid species have been reported from birds [52], three of which are transmitted by *Culicoides* species (*C. stilobezzioides* and *C. travisi*) [52,53]. Future studies examining filarial parasites in birds from areas where infected *Culicoides* occur would help clarify the transmission cycle and host associations of these parasites.

*Leucocytozoon* spp. were the most frequently detected haemosporidian parasites in *Culicoides* spp. from Laos. Based on *cyt b* sequences, two taxa were recognized: *Leucocytozoon caulleryi* and an unidentified *Leucocytozoon* species. Among all known *Leucocytozoon* species, only *L. caulleryi* has been established to be transmitted by *Culicoides*, with *C. arakawae* serving as its principal vector [54,55]. In the present study, *L. caulleryi* was detected in *C. mahasarakhamense*, a species closely related to *C. arakawae* [10]. This finding suggests that *C. mahasarakhamense* may serve as a potential vector of *L. caulleryi* in Laos. The unidentified *Leucocytozoon* lineage has also been detected in *Culicoides* spp. and black flies (Simuliidae) from both Laos [56] and Thailand [33,57], and occurs frequently in domestic chickens [58]. Nevertheless, detection of parasite DNA in hematophagous insects alone does not demonstrate vector competence or natural transmission [23,24]. Experimental transmission studies are therefore needed to determine whether this parasite is biologically transmitted by *Culicoides* spp.

*Plasmodium* sp. was the second most common haemosporidian parasite detected in *Culicoides* spp. Comparison of *cyt b* sequences with those available in GenBank indicated that all positive specimens belonged to *Plasmodium juxtanucleare*, an avian malaria parasite commonly infecting chickens and other birds. Because mosquitoes are the recognized biological vectors of *Plasmodium* spp., the presence of *P. juxtanucleare* DNA in *Culicoides* spp. may well reflect parasite acquisition during blood feeding rather than active transmission. Nevertheless, several *Culicoides* species, including *C. festivipennis*, *C. kibunensis*, *C. pictipennis*, *C. simulator*, and *C. truncorum*, have been experimentally shown to transmit the avian malaria lineage CulPlas1, whereas *C. festivipennis* can also transmit CulPlas2 [59]. Although our findings do not provide evidence that *Culicoides* spp. transmit *P. juxtanucleare* in Laos, they indicate that further investigations into the vector competence of local *Culicoides* species are warranted.

Species of *Haemoproteus* are primarily transmitted by *Culicoides* spp. [60,61]. Two specimens, one *C. similis* and one *C. thurmanae*, were infected with unidentified *Haemoproteus* species. Phylogenetic analyses suggested that these parasites represent two distinct taxa. The *Haemoproteus* lineage detected in *C. similis* clustered with unidentified lineages previously detected in a variety of wild birds and shared an identical *cyt b* sequence with a parasite reported from the spotted munia (*Lonchura punctulata*) in India [39], indicating that they are likely the same species. In contrast, the parasite detected in *C. thurmanae* formed a separate clade with unidentified *Haemoproteus* lineages infecting diverse wild birds. These findings suggest that *C. similis* and *C. thurmanae* are candidate vectors of these avian *Haemoproteus* parasites.

Neither *Leishmania* nor *Trypanosoma* was detected in *Culicoides* specimens investigated in this study. The lack of detection may reflect a low prevalence of these parasites in the sampled areas, the number and geographic coverage of specimens examined, temporal variation in parasite circulation, and limited contact between the sampled *Culicoides* spp. and infected vertebrate hosts. In addition, although *Trypanosoma* and *Leishmania* DNA has been detected in some *Culicoides* spp. elsewhere [18,20,21,22], the vector competence and epidemiological importance of most *Culicoides* spp. for these parasites remain uncertain. Thus, our negative results should not be interpreted as evidence that these parasites are absent from *Culicoides* populations in Laos.

## 5. Conclusions

This study demonstrates that *Culicoides* species in Laos harbor a diverse assemblage of parasites, including filarial nematodes of the genus *Onchocerca* and haemosporidian parasites belonging to the genera *Leucocytozoon*, *Plasmodium*, and *Haemoproteus*. Although molecular detection of parasite DNA in hematophagous insects does not confirm vector competence or natural transmission [23,24], it does provide valuable information for identifying candidate vector species and prioritizing future epidemiological investigations. Widespread and abundant species such as *C. mahasarakhamense*, *C. huffi*, and *C. guttifer* deserve particular attention in future vector competence studies. Our results also highlight the importance of integrating morphological identification with DNA barcoding to achieve accurate species identification. Such an integrative approach is especially critical in studies of vector–parasite associations, where reliable identification of both vectors and parasites forms the foundation for understanding disease transmission.

## Figures and Tables

**Table 1:** Number of female specimens of each *Culicoides* species and parasites detected in Laos.

Subgenus/Species	*n* (Bloodfed)	Filarial Parasite (*n*)	Heamosporidian Parasite (*n*)		
			*Haemoproteus*	*Leucocytozoon*	*Plasmodium*
*Avaritia* Fox					
*C. actoni* Smith	18 (11)	*Onchocerca* sp. (1)	-	*Leucocytozoon* sp. (1)	-
*C. brevipalpis* Delfinado	3 (2)	-	-	*Leucocytozoon* sp. (1)	-
*C. fulvus* Sen & Das Gupta	5 (0)	-	-	*Leucocytozoon* sp. (1)	-
*C. hui* Wirth & Das Gipta	2 (0)	-	-	-	-
*C. jacobsoni* Macfie	2 (0)	-	-	-	-
*C. orientalis* Macfie	5 (1)	-	-	*Leucocytozoon* sp. (1)	-
*Hoffmania* Fox					
*C. innoxius* Sen & Das Gupta	1 (1)	-	-	-	-
*C. peregrinus* Kieffer	39 (36)	-	-	*Leucocytozoon* sp. (1)	*P. juxtanucleare* (1)
*C. sumatrae* Macfie	6 (0)	-	-	*Leucocytozoon* sp. (2)	-
*Meijerehelea* Wirth & Hubert					
*C. arakawae* (Arakawa)	2 (1)	-	-	*Leucocytozoon* sp. (1)	*P. juxtanucleare* (2)
*C. guttifer* de Meijere	7 (5)	*Onchocerca* sp. (2)	-	*Leucocytozoon* sp. (1)	*P. juxtanucleare* (4)
*C. mahasarakhamense* Pramual et al.	25 (16)	*Onchocerca* sp. (1)	-	*Leucocytozoon* sp. (9)	*P. juxtanucleare* (10)
				*L. caulleryi* (1)	
*Monoculicoides* Khalaf					
*C. homotomus* Khalaf	6 (0)	-	-	-	-
*Remmia* Glukhova					
*C. oxystoma* Kieffer	17 (12)	-	-	-	-
*Trithecoides* Wirth & Hubert					
*C. flavescens* Macfie	18 (0)	-	-	*Leucocytozoon* sp. (2)	-
*C. fordae* Lee	3 (0)	-	-	-	-
*C. palpifer* Das Gupta & Ghosh	9 (1)	-	-	*Leucocytozoon* sp. (2)	-
*C. parahumeralis* Wirth & Hubert	10 (3)	-	-	*Leucocytozoon* sp. (1)	-
Subgenus unplaced, *Clavipalpis* species group					
*C. clavipalpis* Mukerji	4 (3)	-	-	*Leucocytozoon* sp. (2)	*P*. *juxtanucleare* (1)
*C. huffi* Causey	26 (13)	*Onchocerca* sp. (2)	-	*Leucocytozoon* sp. (7)	*P*. *juxtanucleare* (9)
*C. similis* Carter, Ingram & Macfie	1 (1)	-	*Haemoproteus* sp. (1)	-	-
Subgenus unplaced, *Ornatus* species group					
*C. pampangensis* Delfinado	2 (0)	-	-	*Leucocytozoon* sp. (1)	-
Subgenus unplaced, *Shermani* species group					
*C. geminus* Macfie	12 (7)	-	-	-	-
*C. thurmanae* Wirth & Hubert	1 (0)	-	*Haemoproteus* sp. (1)	*Leucocytozoon* sp. (1)	-
Subgenus unplaced, *Shortti* species group					
*C. shortti* Smith & Swaminath	20 (12)	-	-	*Leucocytozoon* sp. (2)	-
Unknown species group					
*Culicoides* sp. 1	3 (0)	-	-	*Leucocytozoon* sp. (2)	-
*Culicoides* sp. 2	1 (1)	-	-	*Leucocytozoon* sp. (1)	-
Total	237 (126)	*Onchocerca* sp. (6)	*Haemoproteus* sp. (2)	*Leucocytozoon* sp. (39) *L. caulleryi* (1)	*P. juxtanuleare* (27)

**Table 2:** The top hit sequence from the NCBI GenBank BLAST search results for the 12S rRNA sequences of filarial parasites detected in *Culicoides* species from Laos.

*Culicoides* Species	n	Top Hit (% Similarity)	Accession Number	Host	Country
*C. actoni*	1	*Onchocerca lienalis* (98%)	AY462925	Cattle	United Kingdom
*C. guttifer*	2	*Onchocercidae* sp. (99–100%)	OQ658729	*C. mahasarakhamense*	Thailand
*C. huffi*	2	*Onchocercidae* sp. (99%)	OQ658729	*C. mahasarakhamense*	Thailand
*C. mahasarakhamense*	1	*Onchocercidae* sp. (99%)	OQ658729	*C. mahasarakhamense*	Thailand

**Table 3:** The top hit sequence from the NCBI GenBank BLAST search results for the *cyt b* sequences of *Leucocytozoon* detected in *Culicoides* species from Laos.

*Culicoides* Species	*n*	Top Hit (% Similarity)	Accession Number	Host	Country
*C. actoni*	1	*Leucocytozoon* sp. (99%)	KT290945	Chicken	Malaysia
*C. arakawae*	1	*Leucocytozoon* sp. (99%)	KT290945	Chicken	Malaysia
*C. brevipalpis*	1	*Leucocytozoon* sp. (99%)	KT290945	Chicken	Malaysia
*C. clavipalpis*	2	*Leucocytozoon* sp. (99%)	KT290945	Chicken	Malaysia
*C. flavescens*	2	*Leucocytozoon* sp. (99%)	KT290945	Chicken	Malaysia
*C. fulvus*	1	*Leucocytozoon* sp. (98%)	KT290945	Chicken	Malaysia
*C. guttifer*	1	*Leucocytozoon* sp. (99%)	KT290939	Chicken	Malaysia
*C. huffi*	1	*Leucocytozoon* sp. (99%)	KT290939	Chicken	Malaysia
*C. huffi*	4	*Leucocytozoon* sp. (99%)	KT290945	Chicken	Malaysia
*C. huffi*	2	*Leucocytozoon* sp. (99%)	OP227015	*Culicoides* sp.	Thailand
*C. mahasarakhamense*	9	*Leucocytozoon* sp. (99–100%)	KT290939, KT290944, KT290945, OP227015	Chicken, *Culicoides* sp.	Malaysia, Thailand
*C. mahasarakhamense*	1	*Leucocytozoon caulleryi* (99%)	LC713400	Chicken	Thailand
*C. orientalis*	1	*Leucocytozoon* sp. (99%)	KT290945	Chicken	Malaysia
*C. palpifer*	2	*Leucocytozoon* sp. (99%)	KT290945	Chicken	Malaysia
*C. pampangensis*	1	*Leucocytozoon* sp. (99%)	KT290945	Chicken	Malaysia
*C. parahumeralis*	1	*Leucocytozoon* sp. (99%)	KT290945	Chicken	Malaysia
*C. peregrinus*	1	*Leucocytozoon* sp. (99%)	KT290945	Chicken	Malaysia
*C. shortti*	2	*Leucocytozoon* sp. (99%)	KT290945	Chicken	Malaysia
*C. sumatrae*	2	*Leucocytozoon* sp. (99%)	KT290945	Chicken	Malaysia
*C. thurmanae*	1	*Leucocytozoon* sp. (99%)	KT290945	Chicken	Malaysia
*Culicoides* sp. 1	2	*Leucocytozoon* sp. (99%)	KT290945	Chicken	Malaysia
*Culicoides* sp. 2	1	*Leucocytozoon* sp. (99%)	KT290945	Chicken	Malaysia

**Table 4:** The top hit sequence from the NCBI GenBank BLAST search results for the *cyt b* sequences of *Plasmodium* detected in *Culicoides* species from Laos.

*Culicoides* Species	*n*	Top Hit (% Similarity) (Accession No.)	Accession Number	Host	Country
*C. arakawae*	2	*P. juxtanucleare* (99%)	KU248841	Chicken	Thailand
*C. clavipalpis*	1	*P. juxtanucleare* (99%)	KU248842	Chicken	Thailand
*C. guttifer*	3	*P. juxtanucleare* (99%)	KU248841	Chicken	Thailand
*C. huffi*	9	*P. juxtanucleare* (99%)	KU248841	Chicken	Thailand
*C. mahasarakhamense*	10	*P. juxtanucleare* (99%)	KU248842	Chicken	Thailand
	1	*P. juxtanucleare* (99%)	KU248841	Chicken	Thailand
*C. peregrinus*	1	*P. juxtanucleare* (99%)	KU248842	Chicken	Thailand

**Table 5:** The top hit sequence from the NCBI GenBank BLAST search results for the *cyt b* sequences of *Haemoproteus* spp. detected in *Culicoides* species from Laos.

*Culicoides* Species	*n*	Top Hit (% Similarity)	Accession Number	Host	Country
*C. similis*	1	*Haemoproteus* sp. (100%)	MF565826	*Lonchura punctulata*	India
*C. thurmanae*	1	*Haemoproteus* sp. (97%)	KF192999	*Culicoides* sp.	Germany

## Data Availability

The sequences generated in this study were deposited in NCBI GenBank under accession numbers PZ798657-PZ798680, PZ809999-PZ810067, and PZ815588-PZ815593. All other data and materials supporting this article are reported within the paper.

## Supplementary Materials

The following supporting information can be downloaded at: https://www.mdpi.com/article/10.3390/insects17090963/s1, Data S1.
